# Capacity of ChatGPT to Identify Guideline-Based Treatments for Advanced Solid Tumors

**DOI:** 10.7759/cureus.37938

**Published:** 2023-04-21

**Authors:** Brian Schulte

**Affiliations:** 1 Medicine, University of California San Francisco, San Francisco, USA

**Keywords:** targeted therapy, immunotherapy, chemotherapy, cancer, nccn guidelines, oncology, chatgpt

## Abstract

Background: ChatGPT, created by OpenAI, is a large language model which has become the fastest growing consumer application in history, recognized for its expansive knowledge of varied subjects. The field of oncology is highly specialized and requires nuanced understanding of medications and conditions. Herein, we sought to better qualify the ability of ChatGPT to name applicable treatments for patients with advanced solid cancers.

Methods: This observational study was conducted utilizing ChatGPT. The capacity of ChatGPT to tabulate appropriate systemic therapies for new diagnoses of advanced solid malignancies was ascertained through standardized prompts. A ratio of those medications listed by ChatGPT to those suggested in the National Comprehensive Cancer Network (NCCN) guidelines was produced and called the valid therapy quotient (VTQ). Additional descriptive analyses of the VTQ and its association with incidence and type of treatment were performed.

Results: Some 51 distinct diagnoses were utilized within this experiment. ChatGPT was able to identify 91 distinct medications in response to prompts related to advanced solid tumors. The overall VTQ is 0.77. In all cases, ChatGPT was able to provide at least one example of systemic therapy suggested by the NCCN. There was a weak association between incidence of each malignancy and the VTQ.

Conclusion: The capacity of ChatGPT to identify medications used to treat advanced solid tumors indicates a level of concordance with the NCCN guidelines. As it stands, the role of ChatGPT to assist oncologists and patients in treatment decision making remains unknown. Nonetheless, in future iterations, it may be anticipated that accuracy and consistency in this domain will improve, and further studies will be needed to better quantify its capabilities.

## Introduction

The integration of continuously more advanced forms of machine learning and artificial intelligence (AI) has been an area of controversy within medicine for decades. Similar to now, with utilization of neural networks to categorize electrocardiograms (ECGs) arouse concern regarding accuracy, liability, and capability [1]. The flexibility of these models has allowed for a bevy of new applications including image generation, as well as translation. Leaders within healthcare and technology have sought to assuage these fears, as well as outline means of qualifying abilities of these new tools [2-4].

Within the last few months, ChatGPT has garnered attention through nearly all domains of society, inspiring individuals from multiple fields, medicine included, to probe its flexibility and aptitude at a wide range of tasks [5-6]. ChatGPT, produced by OpenAI, is a large language model (LLM) released for public use in November 2022. LLMs utilize algorithms in conjunction with large amounts of text data to establish probabilities associated words in a given context [7]. The implication is that with proper implementation, and data, LLMs may therefore generate unique sentences based on available information. ChatGPT itself rests on an LLM produced by OpenAI called GPT3.5. GPT3.5 has been sculpted utilizing text data from the internet in coordination with supervised learning and other methods.

In this study, we evaluate the performance of ChatGPT in its ability to make guideline-based treatment recommendations for patients with metastatic solid tumors. Uniformly, the National Comprehensive Cancer Network (NCCN) guidelines, are utilized as a reference standard to ascertain approval by CMS and other insurance organizations as well as determine the expected treatment pathway for patients in defined categories [8]. Understandably, individual patients will vary in their specific clinical circumstances and the ability of a clinician to adjust to nuance is paramount. Nonetheless, most patients with metastatic disease will receive systemic therapy recommendations in line with established guidelines for their diagnosis. Given the degree of standardization within this framework, it makes a suitable environment in which to assess the recommendations of a LLM, such as ChatGPT.

## Materials and methods

Large language model

ChatGPT is a LLM that can respond to complex questions or commands [9]. This model has been honed utilizing reinforcement Learning from Human Feedback. Model responses are assessed for quality by Human AI trainers, with utilization of an incentive structure towards those ranked highest. Proximal policy optimization was then used to fine-tune the model further [10]. The model, in response to queries, can produce varied responses. These have not been qualified for their accuracy within the field of oncology.

Input source

**Figure 1:**
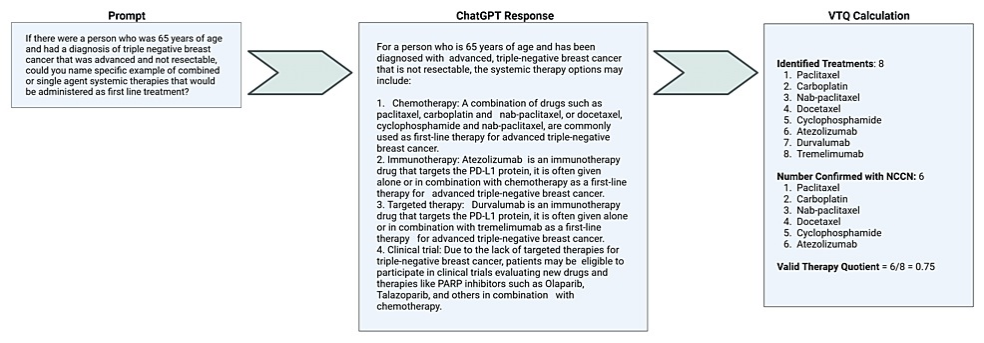
Schematic for query of ChatGPT as well as calculation of VTQ. VTQ, valid therapy quotient; ChatGPT, chat generative pre-trained transformer; NCCN, National Comprehensive Cancer Network

Some 51 different prompts were performed utilizing 32 separate subtypes of solid cancers as defined by NCCN in the first line treatment setting. All sample prompts were standardized to avoid specific reference to gender, or socioeconomic status. An age of reflective of the diagnosis was provided for all questions. The exact prompt as specified for estrogen receptor, progesterone receptor, and HER2 negative breast cancer may be seen in Figure 1. Multiple iterations established that this verbiage was most likely to solicit a suitable response for the purposes of this study. To reduce variability and ensure collection of data, all questions were entered in a single chat session on 1/17/2023 by one user.

Assessment

Outputs from ChatGPT were scored for accordance with NCCN guidelines. Where multiple systemic therapies were suggested, zero points were assigned for suggestions of unlisted therapies. Therefore, only positive scores were possible. The number of valid options was subsequently divided by the total proffered. This determined the valid therapy quotient (VTQ), a rough surrogate for fraction of acceptable recommendations. VTQs range from 0 to 1. VTQ was calculated on a per agent basis, such that combination regimens of multiple agents would be divided into their constituent chemotherapies. Agents withdrawn within the last two years were counted as valid, as it is speculated that ChatGPT was trained using data from 2021. This affected few VTQs. One example includes atezolizumab, which was voluntarily withdrawn by Genentech for patients with triple negative breast cancer. Vague statements such as names of families or types of agents were not counted towards the VTQ.

Descriptive statistics were performed in order to assess associations between types of agents tabulated as first line therapies and accuracy. VTQs were later analyzed within the contexts of families of mechanisms of actions, as well as plotted against predicted annual United States incidence of each disease state when available. A Pearson’s correlation coefficient was calculated assessing the relationship of VTQ to annual incidence [11]. Predicted incidences were extracted from recently published estimates for 2023 [12].

## Results

In total, 51 separate queries were performed for unique clinical situations and 30 distinct histologic subtypes. Examples of 91 medications were provided across all responses (Table 1). Amongst those medications, 58 were classified as targeted therapies, 25 as cytotoxic, and 8 as immunotherapy. The fewest medications offered as possibilities for a prompt was 2, while the highest was 10. Responses returned a mean of 5.8 therapies, with 4.5 of those being listed within the NCCN.

**Table 1:** Medications identified by ChatGPT.

Immunotherapy	Cytotoxic	Targeted
Durvalumab	Gemcitabine	Abemaciclib
Atezolizumab	5-Fluorouracil	Abiraterone
Avelumab	Albumin-bound paclitaxel	Adagrasib
Cemiplimab	Bleomycin	Ado-trastuzumab emtasine
Ipilimumab	Cabazitaxel	Afatinib
Nivolumab	Capecitabine	Alectinib
Pembrolizumab	Carboplatin	Anastrozole
Tremelimumab	Cisplatin	Androgen deprivation therapy
	Cyclophosphamide	Bevacizumab
	Docetaxel	Cabozantinib
	Doxorubicin	Ceritinib
	Etoposide	Cetuximab
	Folinic acid	Crizotinib
	Ifosfamide	Dabrafenib
	Irinotecan	Dacomitinib
	Liposomal doxorubicin	Degarelix
	Mitomycin	Encorafenib
	Oxaliplatin	Entrectinib
	Paclitaxel	Enzalutamide
	Pemetrexed	Erdafitinib
	Temozolomide	Erlotinib
	Topotecan	Everolimus
	Trabectedin	Exemestane
	Vinblastine	Fulvestrant
	Vinorelbine	Goserelin
		GSK2831781
		Imatinib
		Iodine-131
		Lanreotide
		Lapatinib
		Larotrectinib
		Lenvatinib
		Letrozole
		Leuprolide
		Lu 177 dotatate
		Neratinib
		Octreotide
		Osimertinib
		Palbociclib
		Panitumumab
		Pazopanib
		Pertuzumab
		Pralsetinib
		Prednisone
		Ramucirumab
		Regorafenib
		Ribociclib
		Selitrectinib
		Selpercatinib
		Sonidegib
		Sorafenib
		Sotorasib
		Sunitinib
		Trametinib
		Trastuzumab
		Vandetinib
		Vemurafenib
		Vismodegib

**Figure 2:**
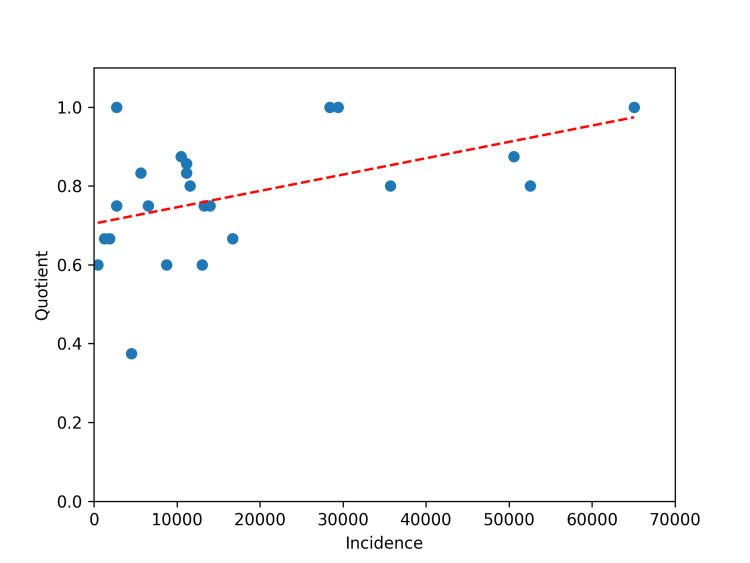
Scatter plot of VTQ by incidence of diagnosis. VTQ, valid treatment quotient

The overall VTQ was 0.77. The range of VTQs spanned from 0.33 to 1.0, indicating that in all circumstances, the model was capable of naming therapies that may be given as first line treatment for advanced or metastatic solid tumors as determined by the NCCN (Table 2). The most common VTQ was 1.0, occurring in 15 separate instances. There was seen to possibly be a weak correlation between incidence and VTQ, with a Pearson’s correlation coefficient of 0.49 (Figure 2).

**Table 2:** Valid treatment quotient by diagnosis. Diagnoses queried with the corresponding total number of treatments named by ChatGPT. Those identified by ChatGPT were compared to the NCCN guidelines in order to determine the total proportion that was valid, or the VTQ. VTQ, valid treatment quotient; NCCN, National Comprehensive Cancer Network

Diagnosis	Total listed	NCCN confirmed	VTQ
Ovarian endometrioid carcinoma	4	3	0.75
Endometrial carcinoma	5	3	0.6
Anal squamous cell carcinoma	6	4	0.67
Basal cell carcinoma	2	2	1
Urothelial carcinoma	6	4	0.67
Hormone receptor positive breast adenocarcinoma	9	9	1
HER2 Positive, hormone receptor negative breast adenocarcinoma	5	3	0.6
Triple negative breast adenocarcinoma	8	6	0.75
Cervical squamous cell carcinoma	4	3	0.75
Colonic adenocarcinoma	10	8	0.8
Esophageal squamous cell carcinoma	6	5	0.83
Esophageal adenocarcinoma	8	7	0.88
Gastric adenocarcinoma	7	6	0.86
Gastrointestinal stromal tumor	3	1	0.33
Head and neck squamous cell carcinoma	5	4	0.8
Hepatocellular carcinoma	3	3	1
Cholangiocarcinoma	8	3	0.38
Clear cell renal cell carcinoma	6	5	0.83
Melanoma (BRAFV600E negative)	4	3	0.75
Melanoma (BRAFV600E positive)	7	7	1
Merkel cell carcinoma	4	4	1
Mesothelioma	5	4	0.8
Well differentiated neuroendocrine carcinoma	6	6	1
Poorly differentiated neuroendocrine carcinoma	4	3	0.75
Non-small cell lung cancer wildtype	6	6	1
Non-small cell lung cancer EGFR exon 19 or 21 mutated	8	8	1
Non-small cell lung cancer EGFR exon 20 mutated	7	7	1
Non-small cell lung cancer ALK fusion positive	4	4	1
Non-small cell lung cancer ROS1 rearranged	3	3	1
Non-small cell lung cancer BRAF V600 mutated	5	5	1
Non-small cell lung cancer NTRK fusion positive	5	4	0.8
Non-small cell lung cancer KRAS G12C mutated	9	6	0.67
Non-small cell lung cancer RET rearranged	7	7	1
Non-small cell lung cancer ERBB2 mutated	8	5	0.63
Non-small cell lung cancer PD-L1 >50%	5	5	1
Non-small cell lung cancer PD-L1 <50%	4	3	0.75
Pancreatic adenocarcinoma	8	7	0.88
Prostatic adenocarcinoma	10	8	0.8
Small cell lung cancer	6	3	0.5
Non-seminomatous germ cell tumor, poor risk	5	4	0.8
Non-seminomatous germ cell tumor, good risk	6	4	0.67
Seminoma, good risk	2	2	1
Differentiated thyroid carcinoma	4	3	0.75
Medullary thyroid carcinoma RET mutated	8	6	0.75
Medullary thyroid carcinoma RET unmutated	9	4	0.44
Anaplastic thyroid carcinoma BRAF mutated	4	2	0.5
Anaplastic thyroid carcinoma BRAF unmutated	8	5	0.63
Dermatofibrosarcoma protuberans	3	2	0.67
Penile cancer	5	3	0.6
Pleomorphic sarcoma	6	4	0.67
Kaposi sarcoma	5	2	0.4

 

Exploratory analyses sought to further characterize families of systemic treatment which may be given. Medications were separated into three broad categories: cytotoxic chemotherapy, targeted therapy, and immunotherapy. While the precise definitions of each remain controversial, authors followed modern conventions in referencing current literature as well as definitions provided by the NCI. Medications which were not so easily classified included folinic acid, and prednisone, which were placed in cytotoxic and targeted therapies, respectively. The VTQ based on drug category was: 0.85 for cytotoxic chemotherapy, 0.68 for targeted therapy, and 0.77 for immunotherapy. ChatGPT also suggested clinical trials in 20 of the queries. ChatGPT mentioned poly-ADP ribose polymerase (PARP) inhibitors in 16 of 20 (80%) instances of reference to clinical trials.

Interestingly, the ChatGPT only identified one medication that was not approved or studied for an oncologic indication, GSK2831781. There were multiple instances of medications identified but given alternative names, such as BLU-667 (pralsetinib), AMG 510 (sotorasib), and MRTX849 (adagrasib).

## Discussion

At the time of writing, this study represents the first descriptive analysis of the capacity of ChatGPT to identify options for systemic treatment in advanced solid tumors. The objective of this study was to better qualify the accuracy, as measured by VTQ, of the named treatments as it pertains to national guidelines, namely the NCCN. With an overall VTQ of 0.77, ChatGPT has shown the capacity to identify medications that may be used within the first line setting of treatment for multiple solid tumors. In total, 91 medications are mentioned, including many that were approved within the window in which ChatGPT was produced. Targeted, immunotherapy, and cytotoxic regimens were referenced. Although beyond the scope of this study, this model appeared to correctly associate medications with these families in multiple responses. Also notable was the fact that only one medication not studied or approved for an oncologic indication was included.

There were other peculiar aspects to some of the responses which included alternate or preliminary drug names, such as Blu-667 for pralsetinib, amongst others. These again, were rare, and may be a result of the dataset which has been employed as part of the LLM. Also intriguing was the incidence of recommendations for consideration of clinical trial for PARP inhibition in 16 cases. Notably, this was in response to contiguous questions, and given that these results were produced in a single session this may be more so a result of vicissitudes in the interface and LLM, and could be unique to this ChatGPT instance.

Recent literature has indicated that ChatGPT may have the ability to pass modern licensing examinations, while other models have conquered complex communication-oriented strategy games [6, 13]. It is easy to speculate the possibilities of these models to assist in, and possibly, provide direction in patient care. While other groups have described the ability of ChatGPT to interface and answer patient-oriented questions, it is important to take its accuracy in doing so in context [14].

**Figure 3:**
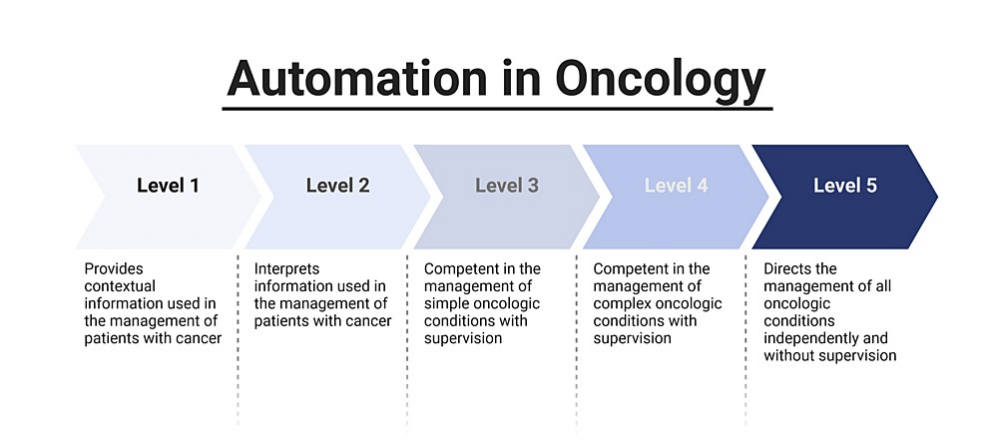
Levels of automation for the care of patients with oncologic diagnoses.

As the field moves forward rapidly, it may be useful to establish a system in which the capabilities of these models may be used. Within medical education, the “reporter,” “interpreter,” “manager,” and “educator” framework (RIME) has been developed in order to describe the progression of students and trainees [15]. Utilizing that system, individuals are observed providing to escalating levels of capacity in the care of patients. While inexact, and certainly a different subject matter, it bears consideration to outline similar definitions for LLMs that could be utilized in the medical space. This has already been considered for other forms of automation [16]. A proposed system is included in Figure 3.

While hypothesis generating, there are clear limitations with the merits of this brief evaluation. Firstly, while the NCCN is one of the more prominent organizations that establishes national guidelines. Other societies within North America likewise publish expert consensuses and the NCCN likewise is limited simply to the United States [17]. International groups, have recommendations which individually may be variably consistent with what has been reported here [18]. Furthermore, all queries were performed by one user in a single session in January 2023. There are known complications that might arise from extended, and repeat questions, which has led to limitations of other LLM tools [19]. Recent changes to the LLM have been performed which may cause different results [20]. The VTQ as a metric also does not account for the many combination regimens given to patients with cancer, which are increasingly frequent. Furthermore, the prompts given did not meaningfully account for many of the nuances of cancer care such as comorbid conditions, prior therapy, dose alteration, and the values and goals of patients. Nonetheless, we feel that this observational study might provide a basis for future utilization of automated systems within the field of oncology.

## Conclusions

ChatGPT was able to identify medications used in first line treatment of advanced solid tumors with a high degree of validity, as measured by the VTQ. There was a numerically higher proportion of incorrectly provided examples for targeted treatments. While there remain some areas of improvement, iteration on the current model will likely continue to better accuracy and comprehensiveness. Additional studies should be performed in order to longitudinally assess the capabilities of ChatGPT and other LLMs in identifying appropriate therapies for patients with advanced malignancies.
